# Artificial Intelligence in Paediatric and Adolescent Fracture Detection: A Systematic Review and Meta-Analysis

**DOI:** 10.7759/cureus.92199

**Published:** 2025-09-13

**Authors:** Jordan Calleja, Kyle Muscat, Jacques Calleja, Gregory Firth

**Affiliations:** 1 General Surgery, Mater Dei Hospital, Msida, MLT; 2 General Surgery, The Grange University Hospital, Cwmbran, GBR; 3 Trauma and Orthopaedics, Barts Health NHS Trust, London, GBR

**Keywords:** artificial intelligence, diagnostic accuracy, fracture, fracture detection, imaging, meta-analysis, paediatric, systematic review

## Abstract

Fractures are among the most common injuries in children, yet their radiographic detection is challenging due to the unique anatomy of the developing skeleton, leading to significant diagnostic errors. To address this, a systematic review and meta-analysis was conducted to evaluate how accurately and efficiently artificial intelligence (AI) detects fractures in children and adolescents. Following PRISMA (Preferred Reporting Items for Systematic Reviews and Meta-Analyses) guidelines, a systematic search of PubMed, EMBASE, and Web of Science identified 11 studies published between 2019 and 2024 evaluating AI for detecting appendicular skeletal fractures in patients under 21 years. A meta-analysis revealed that standalone AI demonstrated a statistically significantly higher sensitivity compared to human interpretation (mean difference: 0.04, 95% CI [0.02, 0.07], p = 0.0005) with non-inferior specificity. Furthermore, AI-assisted diagnosis led to a statistically significant improvement in clinician sensitivity (mean difference: 0.07, p = 0.003). To sum up, AI exhibits high diagnostic performance for paediatric fractures and serves as a promising adjunct tool to enhance clinical efficiency and accuracy; however, further large-scale, multi-centre prospective trials are required to validate its real-world applicability and address current limitations before widespread adoption.

## Introduction and background

Fractures represent one of the most common injuries in children presenting to emergency departments, often posing diagnostic challenges due to subtle radiographic findings and the variability of paediatric skeletal anatomy. Artificial intelligence (AI) has demonstrated the potential to improve diagnostic accuracy and efficiency in detecting fractures. This systematic review evaluates the performance of AI algorithms in paediatric fracture detection.

AI is the application of algorithms that provide machines with the ability to solve problems that traditionally require human intelligence. Now a fundamental part of everyday life, AI comprises several subfields, including machine learning, deep learning, and generative AI. Machine learning is a subset of AI that enables machines to learn patterns from data and make decisions without being explicitly programmed. Deep learning, a further subset of machine learning, uses neural networks with multiple layers to analyse complex patterns in data, making it particularly powerful for image and speech recognition. Generative AI, another subfield, focuses on creating new content based on learned patterns, such as generating realistic images, text, and even synthetic medical data to augment AI model training.

Fractures are one of the most common reasons children present to the emergency department, with an estimated 50% of all children sustaining a fracture during childhood. These injuries can be challenging to diagnose due to subtle radiographic findings and variations in skeletal anatomy during growth. In paediatrics, missed fractures are a significant cause of delayed treatment and may lead to long-term disability. This is particularly important due to medicolegal considerations, as studies have found that surgical specialities produce the highest number of malpractice claims, with Orthopaedic Surgery ranking first. Studies indicate that emergency physicians may miss up to 11% of paediatric fractures [1], and missed fractures have been identified as the most common cause of misdiagnosis, accounting for up to 44% of errors [2].

Artificial intelligence in orthopaedics and fracture detection

Advances in computational power, coupled with the increasing availability of large-scale medical datasets, have facilitated the rapid expansion of AI into healthcare. Within the field of orthopaedics, AI has seen widespread application in diagnostic imaging analysis, preoperative risk stratification, and clinical decision support, with the goals of enhancing patient outcomes and optimising clinical workflows. One of the key areas where AI has shown significant promise is in fracture detection. The accurate identification of fractures in children is crucial, as misinterpretations can lead to long-term complications and functional impairment. Paediatric fractures, particularly those involving the appendicular skeleton, can be subtle and challenging to diagnose, often requiring an experienced radiologist for accurate interpretation. However, given the global shortage of paediatric radiology specialists and the increasing demand for rapid diagnostic tools, AI-based systems have emerged as potential adjuncts to traditional radiographic interpretation. The application of AI in fracture detection typically involves convolutional neural networks (CNNs) trained on extensive datasets of radiographic images.

Evidence indicates that these AI systems can achieve diagnostic accuracy on par with, or superior to, human radiologists. Moreover, the integration of AI as a decision support tool has been shown to improve the diagnostic performance of junior clinicians and decrease the time required for interpretation. The National Institute for Health and Care Excellence (NICE) has advocated for the integration of AI into clinical practice to improve fracture detection accuracy [3]. Beyond fracture detection, AI has been implemented in various other aspects of orthopaedic care. It is used in automated image analysis for conditions such as osteoarthritis, scoliosis, and osteoporosis, enabling earlier, more precise diagnoses. AI has also been applied to preoperative planning, where machine learning models can predict the optimal implant size and alignment for joint replacement surgeries [4].

Robotic-assisted surgery, guided by AI algorithms, has been shown to improve the precision of procedures like total knee and hip arthroplasty [5]. AI is also utilised in predictive analytics to identify patients at higher risk of postoperative complications such as infections, thromboembolism, and implant failure. Additionally, natural language processing (NLP) is used to extract clinical insights from unstructured electronic health records, streamlining patient management. In orthopaedic trauma care, AI-based decision support systems help triage patients and optimise resource allocation [6]. 

Despite its potential, the integration of AI into clinical practice presents several challenges. Variability in model performance across different populations, inconsistencies in ground truth labelling, and the lack of standardised validation methodologies remain key barriers to widespread adoption. Furthermore, ethical and medicolegal considerations, such as the transparency of AI decision-making and clinician accountability, must be addressed to ensure safe and effective implementation. This systematic review aims to evaluate the current literature on the use of AI in detecting paediatric fractures by summarising its diagnostic performance, advantages, limitations, and potential role in clinical workflows. By critically analysing existing studies, this review will provide insights into the effectiveness of AI-based fracture detection systems and highlight areas for future research.

## Review

Methods

This systematic review was registered with the PROSPERO International Prospective Register of Systematic Reviews (CRD42024619744) and follows the Preferred Reporting Items for Systematic Reviews and Meta-Analyses (PRISMA) guidelines.

Literature Review

A comprehensive search was performed using the PubMed, EMBASE, and Web of Science databases for studies published in English between January 2019 and December 2024. The search used database-specific Boolean strategies with terms and word variations of ‘fracture’, ‘paediatrics’, and ‘Artificial Intelligence’. The eligibility criteria included studies focusing on fracture detection in the appendicular skeleton using plain radiographs in patients aged 0-21 years. Randomised controlled trials, prospective and retrospective cohort studies, and diagnostic accuracy studies that evaluated an AI algorithm and reported quantitative outcomes were included.

**Figure 1:**
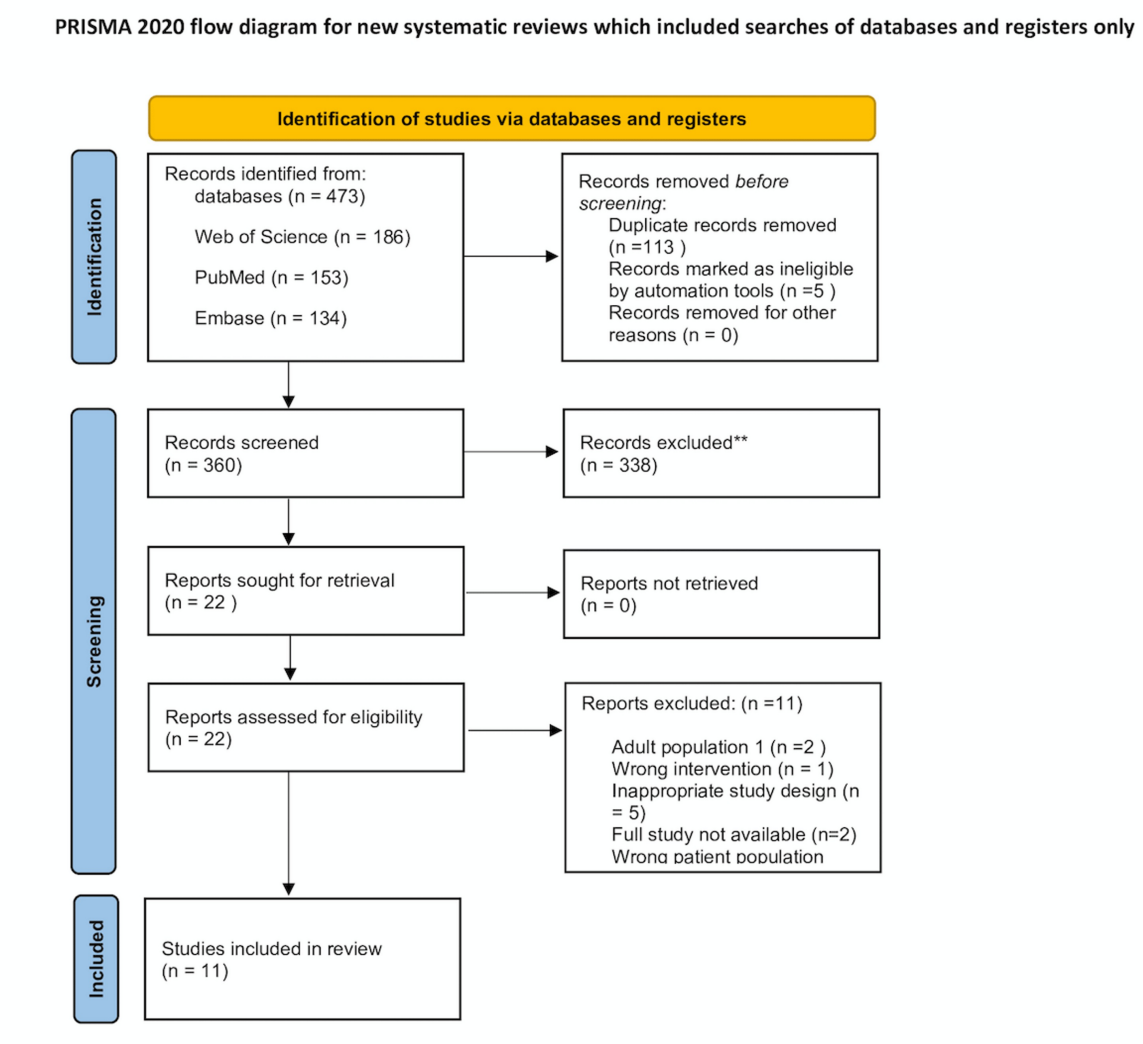
PRISMA chart depicting the selection of studies PRISMA: Preferred Reporting Items for Systematic Reviews and Meta-Analyses

The exclusion criteria comprised studies not written in English; systematic reviews, case reports, editorials, opinion articles, and pictorial reviews; and multimedia files such as online videos and podcasts. Studies were also excluded if the reference standard was unclear, if no distinct paediatric subgroup was identified, if they focused on adults or conditions unrelated to fractures, or if they were qualitative-only studies. Studies employing other imaging modalities, such as CT or ultrasound, or those that were not fully accessible, were also excluded. Two reviewers independently screened all titles and abstracts. A full-text review was then performed for the remaining 22 studies, of which 11 were subsequently excluded (Figure 1). A third reviewer was consulted to resolve any conflicts.

Data from each study - including the AI system used, number of patients and radiographs, participant age, inclusion and exclusion criteria, reference standard, sensitivity, specificity, positive predictive value (PPV), and negative predictive value (NPV) - was entered into a spreadsheet (Microsoft Excel) by a single reviewer. When required, the standard deviation (SD) was calculated from the 95% confidence intervals (CI) for each relevant study. Therefore, the formula, SE = (upper limit - lower limit) / 3.92, was used, then SD = SE √N.

Risk of Bias

A modified QUADAS-2 tool was utilised, using the QUADAS-2 framework combined with the CLAIM checklist to assess risk of bias. This approach ensures a standardised evaluation of diagnostic accuracy studies while addressing AI-specific considerations. The assessment focuses on four domains:

Patient selection: Risk of bias will be assessed by evaluating the representativeness and balance of the patient population, data sources, and inclusion/exclusion criteria. Applicability will consider alignment with real-world paediatric fracture demographics and the AI’s intended usage.

Index test (AI algorithm): Risk of bias includes evaluation of reported performance metrics, uncertainty measures, and algorithm transparency. Applicability will assess external validation and the AI’s generalisability across diverse settings and populations.

Reference standard: Risk of bias will examine the reliability of the “ground truth,” including blinding and clinician expertise. Applicability will ensure the reference standard aligns with accepted clinical practices.

Flow and timing: Bias will be assessed based on consistent application of the index test and reference standard, avoiding temporal discrepancies.

Meta-analysis was performed using Review Manager (RevMan) software [7]. A random-effects model was employed using the inverse variance method with mean difference as the outcome measure, assuming high heterogeneity due to variability in study designs, populations, AI models, and reference standards across the included studies. Forest plots were generated to visualise the data, and funnel plots were used as a visual aid to assess for publication bias.

Results

The systematic search yielded 473 studies, from which 113 duplicates were removed. After title and abstract screening, 338 studies were excluded, leaving 22 full-text articles for evaluation. Following this review, 11 studies that met the inclusion criteria were analysed (Table 1).

**Table 1:** Summary of the included studies AI: artificial intelligence; CNN: convolutional neural network; R-CNN: region-based convolutional neural network

Study	AI system	Body part	No. of radiographs	No of participants	Age, years	Inclusion criteria	Exclusion criteria	Ground truth	Ground truth blinded?	Single centre?
Choi et al., 2020 [8]	Developed a dual-input CNN	Elbow	95	48	Range: 0–19, no median or mean provided, but % in each age group provided in the study	Paeds ED suspected traumatic supracondylar 01/2013 – 12/2018	Not initial radiograph, patients with nonsupracondylar fracture, dislocation, or underlying bone dysplasia	All radiographs were re-reviewed by two paediatric radiologists	Yes	Two centres, same city
Dupuiset al., 2022 [9]	Rayvolve	All (except Radiographs of the skull, ribs, and spine)	2,634 radiographic sets (images = 5,865)	2,549	Mean age, 8.5 ± 4.5	Radiography sets 03/2019-/03/2020 from all consecutive trauma paediatric ED patients	Febrile lameness, whitlow or other infectious context, foreign body detection, or some other indisputably non-traumatic context	Senior radiologist from panel of 11 - including author	Unclear	Yes
Hayashi et al., 2022 [10]	BoneView	Hand/wrist, elbow/upper arm, shoulder/clavicle, foot/ankle, leg/knee	300 (60 per body part)	300	2–21, 10.8 ± 4.9	Post-traumatic X-rays from a US-based data provider	Pelvis, skull, spine, rib cage radiographs, non-acute injury, poor image quality quota reached	Two board-certified MSK radiologists marked a bounding box; cases of disagreement were resolved by a third radiologist	Yes	US data provider
Nguyen et al., 2022 [11]	BoneView	Hand/wrist, elbow/upper arm, shoulder/clavicle, foot/ankle, leg/knee	300	300	10.8 ± 4.9	Anonymised radiographs of- paediatric patient, 2-21 years	Body part not intended for use with BoneView (pelvis, skull, spine, rib cage). Poor quality exams. Poor image quality	Two MSK radiologists; discrepancies were resolved by consensus with a third radiologist	Yes	US data provider
Zech et al., 2022 [12]	Faster R-CNN model	Wrist	125 (test)	395	Mean = 10.1, range: 0.8-17.8	0-18-year-old patients with wrist radiographs	Follow-up imaging, cast or splint	Report by the paediatric fellowship-trained attending radiologist at the time of clinical interpretation was used to establish the ground	No	Yes
Zech et al., 2023 [13]	BoneView	Upper extremity radiographs (finger/hand, wrist/forearm, elbow, humerus, shoulder/clavicle)	819 (internal test)	819	Internal test 10.24; 5.86	All upper extremity radiographic examinations performed on patients aged 0–21 years between 1/1/2015 and 12/31/2021 (n = 44,729 examinations) from initial encounter	Follow-up or bone age examination, ACJ, and scapula, missing location, prior surgery, cast/splint, subacute injury	Silver standard - NLP algorithm from the report. Gold standard - manual review by a resident based on the NLP algorithm, bound box over fracture or elbow effusion. For test data, any discrepancy between the original report and the manual review was decided by a review by the senior author (MSK radiologist with 13 years of experience)	Yes	Yes
Altmann‐Schneider et al., 2023 [14]		Lower limb, elbow, and forearm	2,100 lower limbs, 2,051 forearms, 1,104 elbows	1,000 per body part	Forearm 7.8 ± 3.9. Elbow 7.7 ± 3.7. Lower Limb 4.9±4	Radiographs of either the forearm, lower leg, or elbow in at least two projections were performed on the day of attendance at the emergency department.	Radiographs of patients with fractures with a high specificity for child abuse (e.g., metaphyseal corner fractures) were excluded, as the AI software is not trained to detect the	All radiographs contained an original report from the attending paediatric radiology staff member with varying degrees of experience. Each radiograph received a second reading performed by a certified paediatric radiologist	No	Yes
Gasmi et al., 2023 [15]	Rayvolve	All	878	878	<18, mean. Female 8.4. Male 8.3	<18, recent non-life-threatening injury, and at least one appendicular radiograph	Radiographs not available for review, not reported	2 paediatric radiologists	Yes	Yes
Dupuis et al., 2024 [16]	SmartUrgences	Elbow	741 radiographic sets, 1,601 images	695	7.27 ± 3.97	This single-centre retrospective study was conducted on elbow radiography sets collected from January 2018 to December 2021 from all consecutive patients younger than 18 years who were referred by the paediatric emergency room in a trauma context	Wrong X-ray location, osteitis search, infectious context foreign body search, or no Milvue interpretation	Read by a junior and then a senior radiologist during routine care	No	Yes
Kavak et al., 2024 [17]	CNN model You Only Look Once (YOLO) v8	All	7,150	.	Mean age 8.3 years	Availability of a radiograph of an appendicular part taken after a recent trauma	Radiographs featuring implants, casts, or any other pathological lesions in the bones, as well as patients presenting fractures highly specific to child abuse (e.g., metaphyseal corner fractures), were excluded	Consensus bounding box between 3 radiologists	Unclear	Yes
Zech et al., 2024 [18]	Childfx	Upper extremity	1,693	240	Mean = 11.3 years, range: 0–22	Same as Zech et al. 2023	Same as Zech et al. 2023	MSK radiologist + paediatric radiologist; disagreements were resolved by a second musculoskeletal radiologist	Yes	Yes

**Figure 2:**
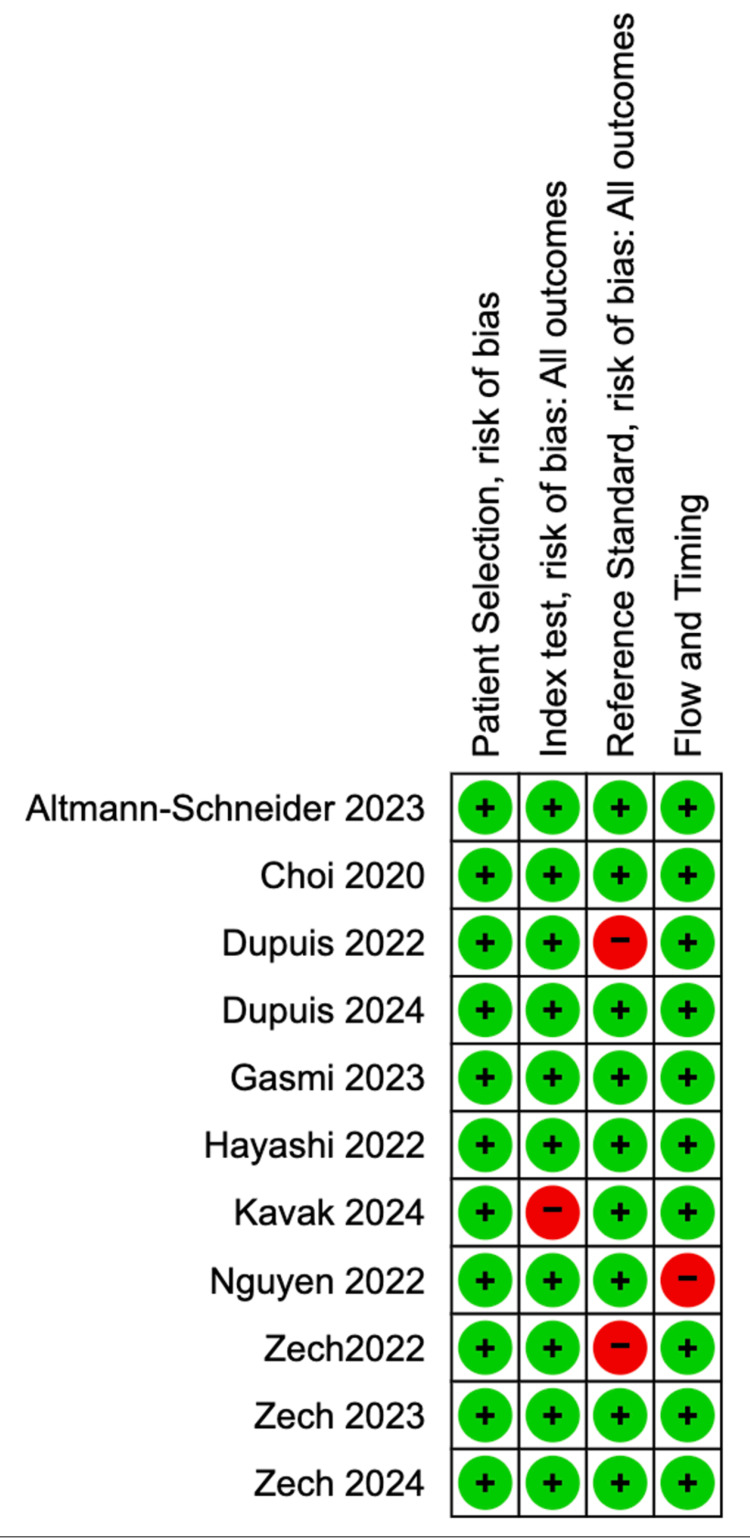
Risk of bias analysis

This review focused on paediatric fractures of the appendicular skeleton. Of the 11 included studies, eight were single-centre. The exceptions were a two-centre study by Choi et al., which was limited to a single city, and studies by Nguyen et al. and Hayashi et al., which used data from a US provider. Several studies excluded radiographs of limbs in a plaster or cast and metaphyseal corner fractures. All included studies used retrospective data, and six directly compared AI performance with human readers. The AI systems evaluated included BoneView (four studies), Rayvolve (two studies), SmartUrgences, YOLO v8, Childfx, a custom CNN, and a faster R-CNN model. In four studies, the ground truth was not blinded to clinical details, introducing a potential source of bias. Risk of bias analysis (Figure 2) identified a high risk of bias for the reference standard in Dupuis 2022 due to the use of a single radiologist's report as the gold standard. The Kavak 2024 study was found to have a high risk of bias in the index test, as the AI algorithm was unable to investigate lateral radiographs, while the Nguyen 2022 study was considered high risk for timing and flow since radiographs were read with AI assistance immediately after being read without it.

AI Diagnostic Accuracy

The overall sensitivity of AI in detecting fractures ranged from 88% to 96% across the included studies (Table 2). This performance varied based on the AI model used, with BoneView studies reporting sensitivity between 80.5% and 92.9%, Rayvolve studies demonstrating sensitivity of approximately 95%, and other models reporting sensitivity ranging from 88% to 96%. Six studies directly compared AI performance with human readers. For example, Nguyen et al. (2022) reported that AI achieved an AUC of 0.932, outperforming all human readers with a stand-alone sensitivity and specificity of 91% and 90%, respectively. Zech et al. (2022) found that AI demonstrated an accuracy of 88%, significantly higher than the residents' accuracy of 80%. Zech et al. (2023) showed that AI sensitivity reached 90.8% in overnight preliminary interpretations. Zech et al. (2024) noted improved AUC scores for both radiology and paediatric residents when assisted by AI, with AI-assisted interpretation reducing radiograph interpretation times from 52.1 seconds to 38.9 seconds (p = 0.030).

**Table 2:** Literature review results AI: artificial intelligence; NPV: negative predictive value; N: not reported; PPV: positive predictive value

Study	Diagnosis	No. of patients	Sensitivity	Specificity	PPV	NPV	Accuracy
Altmann-Schneider et al., 2023 [14]	Lower limb	1,000	90.6 (88.0–92.8)	97.1 (96.1–97.9)	92.6	96.2	NR
	Forearm	1,000	96.0 (94.7–97.1)	92.9 (91.0–94.4)	94.4	94.9	NR
	Elbow	1,000	80.5 (88.7–93.8)	63.7 (59.7–67.6)	69	89.5	NR
Dupuis et al., 2024 [16]	Fracture detection	695	92.9 (89.0−95.5)	76.8 (72.6-80.5)	70.8 (65.9−75)	94.7 (91.7−96)	82.9 (79.9-85.5)
Dupuis et al., 2022 [9]	AI detection	2,634	0.957 (0.94-0.969)	0.912 (0.898-0.925)	NR	NR	0.926 (0.915-0.936)
Nguyen et al., 2022 [11]	AI detection		91	90	NR	NR	93.2
	Human detection	300	73.17 (65.33-80.07)	89.58 (83.55- 93.97)	NR	NR	NR
	AI-assisted	300	82.67 (75.65-88.36)	90.33 (84.43-94.55)	NR	NR	NR
Kavak et al., 2024 [17]	AI detection	5,150	95.8 (95.5-96.6)	72.3 (71.13-73.57)	91.3	84.96	90
Zech et al., 2024 [18]	AI detection	240	90 (83.2-94.7)	88.3 (81.2-93.5)	NR	NR	89.2 (85.2-93.1)
	Human detection (resident)	240	78.1 (72.0-84.1)	0.756 (0.710-0.801)	NR	NR	0.768 (0.730-0.806)
	Human detection (attending)	240	84.2 (78.6-0.897)	0.892 (0.850-0.934)	NR	NR	0.867 (0.832-0.902)
	AI-assisted (resident)	240	87.6 (84.5-0.908)	0.869 (0.827-0.912)	NR	NR	0.876 (0.845-0.908)
	AI-assisted (attending)	240	85.8 (0.802-0.914)	0.921 (0.882-0.959)	NR	NR	0.890 (0.856-0.924)
Zech et al., 2023 [13]	AI detection	819	0.922 (0.896-0.948)	0.866 (0.833-0.899)	0.894 (0.873-0.915)	NR	NR
	Human detection	819	0.87 (0.838-0.903)	0.832 (0.795-0.868)	0.851 (0.827-0.875)	NR	NR
Zech et al., 2022 [ 12]	AI detection	125	88% (78–94%)	89% (76–96)	NR	NR	88 (81–93)
	Human detection (residents)	500	78% (73–82%)	85% (79–90)	NR	NR	80% (77–84)
	AI-assisted (residents)	500	91% (87–94%)	96% (91–98%)	NR	NR	93% (0.9-0.95)
Hayashi et al., 2022 [10]	AI detection	300	91.3% (85.6, 95.3)	90.0% (84.0,94.3)	NR	NR	0.93
Gasmi et al., 2023 [15]	AI detection	878	95.7% (0.93-0.99)	91.6% (0.89–0.94)	74.7% (0.69-0.80)	98.8% (0.98–0.99)	0.79 (0.74-0.83)
Human detection	Paediatric radiologists		98.4% (0.97-1.00)	99.7% (0.99-1.00)	98.9% (0.97-1.00	99.0% (0.99-1.00)	0.98 (0.97-0.99)
	Emergency physicians		81.9% (0.76-0.88)	95.0% (0.93-0.97)	81.0% (0.75-0.87)	95.0% (0.94-0.97)	NR
	Senior residents		95.1% (0.92-0.98)	98.0% (0.96-0.99)	92.5% (0.89-0.96)	98.7% (0.98-1.00)	NR
	Junior residents		90.1% (0.86-0.94)	96.6% (0.95-0.98)	87.2% (0.93-0.92	97.4% (0.96-0.99	NR
Choi et al., 2020 [8]	AI detection	258	93.9% (0.90–0.93)	92.2% (0.874–0.956)	80.5 (71.7–87.1)	97.8 (94.5–99.1)	0.992 (0.947–1.000)
	Radiologist 1	95	95.7 (78.1–99.9)	97.2 (90.3–99.7)	91.7 (73.7–97.7)	98.6 (91.1–99.8)	0.977 (0.924–0.997)
	Radiologist 2	95	95.7 (78.1–99.9)	97.2 (90.3–99.7)	91.7 (73.7–97.7)	98.6 (91.1–99.8)	0.997 (0.956–1.000)
	Radiologist 3	95	95.7 (78.1–99.9)	100.0 (95.0–100.0)	100	98.6 (91.4–99.8)	0.978 (0.924–0.997)
	Radiologist 1 w/AI	95	100.0 (85.2–100.0)	97.2 (90.3–99.7)	92.0 (74.6–97.8)	100	0.993 (0.949–1.000)

**Figure 3:**
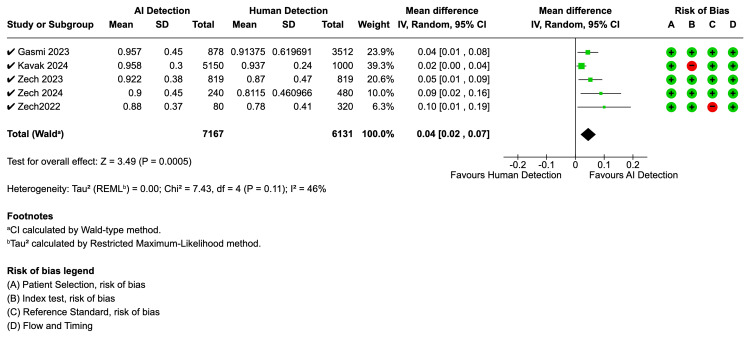
Forest plot for sensitivity - AI vs. human reader AI: artificial intelligence; CI: confidence interval; SD: standard deviation

To synthesise these findings, a meta-analysis was performed. The pooled analysis (Figure 3) of five studies assessing sensitivity revealed a statistically significant improvement in favor of AI, with a mean difference of 0.04 (95% CI: 0.02, 0.07, Z = 3.49, p = 0.0005). This analysis showed moderate heterogeneity between studies (I² = 46%, p = 0.11).

**Figure 4:**
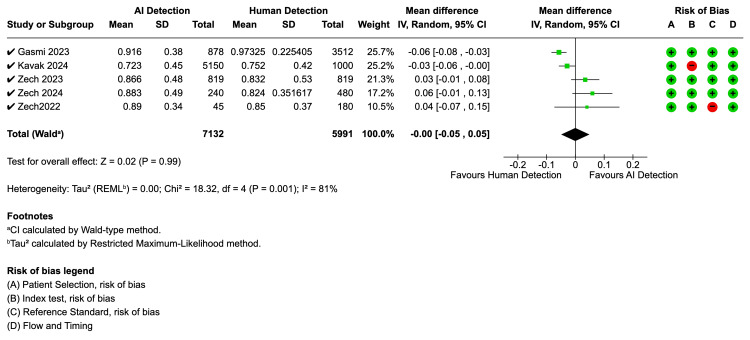
Forest plot for specificity - AI detection vs. human reader AI: artificial intelligence; CI: confidence interval; SD: standard deviation

Specificity ranged widely from 63.7% to 92.2%, with significant variation between studies. BoneView studies reported a specificity between 63.7% and 92.9%. Rayvolve studies reported specificity above 90%. SmartUrgences demonstrated significantly lower specificity for plastered patients at 54.5% compared to 95.5% for uncasted patients. The pooled analysis for specificity (Figure 4), which also included five studies, showed no significant difference between AI and human interpretation, with a mean difference of 0.00 (95% CI: -0.05, 0.05, Z = 0.02, p = 0.99). The specificity analysis exhibited substantial heterogeneity (I² = 81%, p = 0.001).

Anatomic Location and Fracture Type

AI performance varied by anatomical location and fracture type. The study by Altmann-Schneider et al. (2024) divided fractures into lower leg, forearm, and elbow categories. The highest sensitivity and specificity were seen in forearm fractures at 92.9% and 98.1%, respectively. The lowest sensitivity, 87.5%, and the lowest specificity, 80.5%, were observed for lower leg fractures. Fractures such as complete diaphyseal and metaphyseal radius, ulna, and tibia fractures had detection rates between 99% and 100%. More inconspicuous fractures, such as Salter-Harris II fractures of the proximal tibia, showed a detection rate of 60%, bowing fractures of the radius had a detection rate of 18%, and avulsion fractures of the ulnar epicondyle showed a detection rate of 25%.

The study by Zech et al. demonstrated a greater improvement in detecting non-obvious fractures, particularly where fractures were not displaced or angulated (p = 0.001). This improvement was still observed in more obvious cases (p = 0.013). The study by Nguyen also showed a greater improvement in the detection of non-obvious fractures, with the highest gains in diagnostic performance noted in buckle fractures and Salter-Harris II and IV fractures, with absolute differences of 20.63% (p < 0.001), 11.31% (p = 0.003), and 29.17% (p = 0.006), respectively. The study by Dupuis et al. reported the lowest sensitivity for pelvis fractures at 75% and noted that sensitivity for plastered patients was significantly lower at 54.5% compared to 95.5% for patients without casts.

AI-Assisted Interpretation

**Figure 5:**
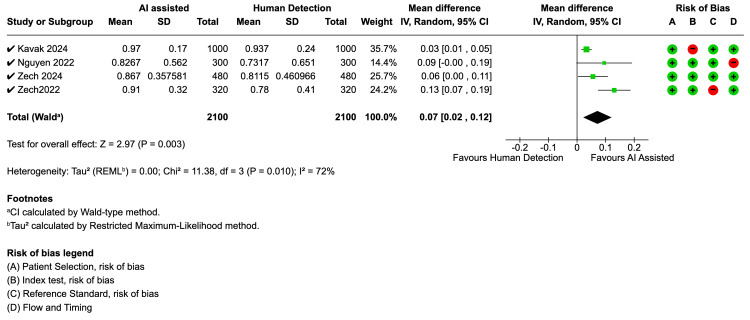
Forest plot for sensitivity - AI-assisted vs. human reader AI: artificial intelligence; CI: confidence interval; SD: standard deviation

**Figure 6:**
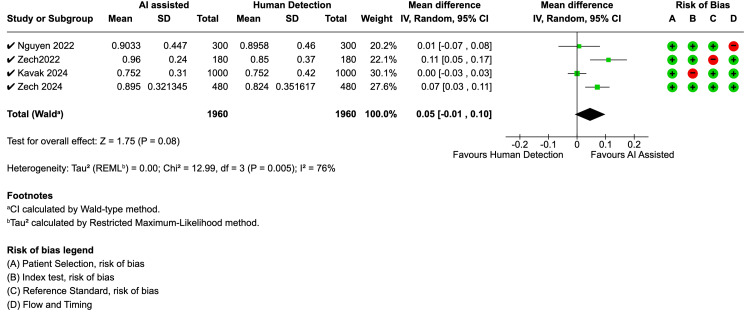
Forest plot for specifity - AI-assisted vs. human reader AI: artificial intelligence; CI: confidence interval; SD: standard deviation

Four studies evaluated the impact of AI-assisted interpretation on clinician performance. Zech et al. (2022) found that AI assistance significantly improved resident accuracy from 80% to 93%, particularly for buckle fractures, while Nguyen et al. (2022) reported a 10% absolute increase in sensitivity across all readers when using AI. The meta-analysis for AI-assisted detection showed a statistically significant improvement in sensitivity (Figure 5), with a mean difference of 0.07 (p=0.003). This analysis, however, showed considerable heterogeneity (I² = 72%, p=0.01). The analysis for specificity (Figure 6) showed a trend towards improvement with a mean difference of 0.05, though this was not statistically significant (p=0.08), and also demonstrated significant heterogeneity (I² = 76%, p=0.005). The study by Zech et al. (2024) was the only one to assess efficiency and found that AI-assisted interpretation also significantly reduced radiograph interpretation times. 

Discussion

This systematic review and meta-analysis provide compelling evidence that AI holds significant promise in the challenging domain of paediatric fracture detection. The pooled data clearly demonstrate that standalone AI systems possess a statistically significantly higher sensitivity compared to human readers, without a corresponding drop in specificity. This finding suggests that AI can identify more true fractures than clinicians alone. While a 4% absolute increase in sensitivity may appear modest, its clinical significance is potentially substantial in high-volume settings like an emergency department. Perhaps more importantly for clinical practice, the analysis also revealed that AI-assisted interpretation leads to a statistically significant improvement in clinicians' own sensitivity.

**Figure 7:**
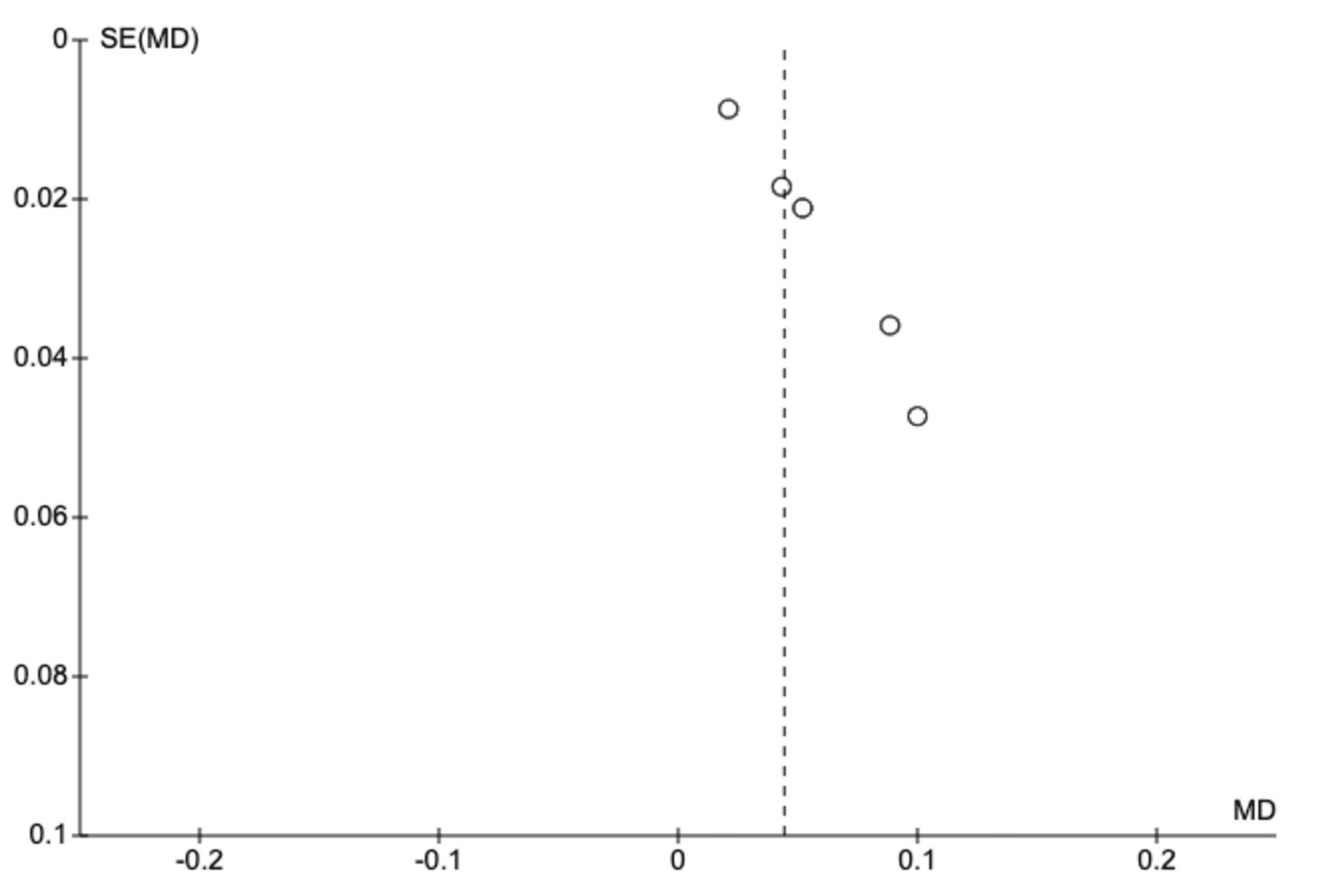
Funnel plot - sensitivity MD: mean difference; SE: standard error

**Figure 8:**
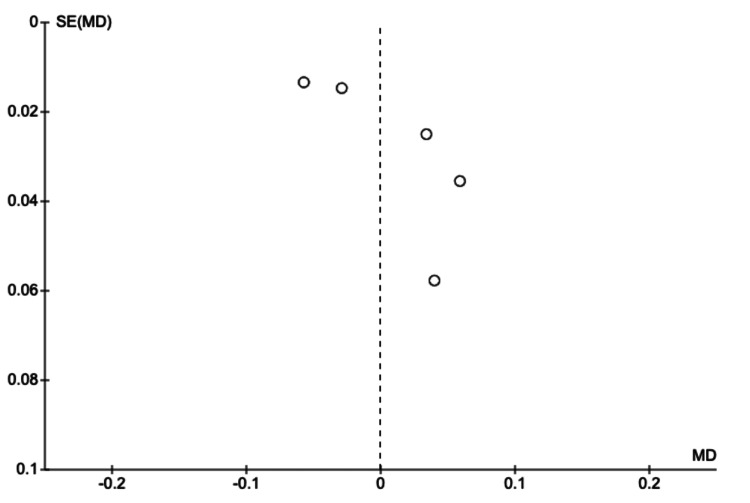
Funnel plot - specificity MD: mean difference; SE: standard error

These results collectively underscore the potential of AI not as a replacement for human expertise, but as a powerful augmenting tool that can enhance diagnostic capabilities. By integrating AI assistance into clinical workflows, clinicians may benefit from an increased ability to detect subtle fractures, thereby reducing the rate of missed injuries, while maintaining the high specificity required to avoid unnecessary interventions.. The publication bias was investigated through the use of funnel plots (Figures 7, 8), which were seen to be symmetrical, showing that there is a limited effect of publication bias in studies included for meta-analysis.

A critical aspect of interpreting these meta-analyses is the consideration of heterogeneity. The analysis of standalone AI specificity and both analyses for AI-assisted interpretation showed considerable to substantial heterogeneity between the included studies. This variability suggests that while the overall trend is positive, the magnitude of AI's effect differs significantly across different contexts. This is likely attributable to the wide diversity in the AI models themselves, the specific patient populations studied, and the methodologies employed in each trial. Therefore, while AI demonstrates high diagnostic accuracy, the limited external validation of many of these models remains a concern, raising questions about their generalisation to broader paediatric populations and varied clinical settings.

There are also concerns regarding data security and the potential ethical implications of AI; however, a survey performed indicated that 64% of parents were comfortable with an AI program diagnosing their child's fracture, while 82% supported AI being used as an adjunct to a clinician’s diagnosis. This suggests that while there is growing trust in AI applications, human oversight remains a crucial factor for widespread acceptance and successful implementation in paediatric fracture detection [19].

Limitations

This systematic review is limited by potential sources of bias and methodological issues. A primary concern is the overlap in authorship and patient cohorts in several studies, which could skew results and reduce the generalisability of the findings. Bias may also be present in the meta-analysis, as studies focusing on specific anatomical locations might report higher sensitivity than those examining all fracture types. The funding of included studies by the AI software companies creates a potential conflict of interest and a significant risk of bias, which must be considered when interpreting data. Additionally, all included studies were retrospective, which inherently limits the ability to establish causality or control for confounding variables. The scope and design of the included studies also present constraints. Most were single-centre studies with small external test datasets, and each assessed only one AI software, making it impossible to compare the performance of different systems.

The review highlights a critical need for multicentre randomised controlled trials that use a "gold standard" reference like CT imaging for fracture confirmation. Furthermore, the exclusion of crucial clinical information, such as patient history and physical exams, from the diagnostic process is a major weakness that could lead to misclassification. High heterogeneity was noted, likely reflecting the diversity in AI models and patient populations. Finally, the inconsistent distribution of fracture types meant that less obvious injuries, like buckle or Salter-Harris fractures, were more likely to be missed, affecting overall accuracy.

## Conclusions

AI demonstrates high diagnostic performance in detecting paediatric fractures, with a statistically significantly higher sensitivity than human readers. When used as an assistive tool, it significantly improves clinicians' detection rates. Future research should prioritise multi-centre, prospective, randomised controlled trials to evaluate AI performance across diverse clinical settings, enhancing the generalisability of findings. Comparative studies involving multiple AI systems and the integration of clinical data are essential to determine the most effective and cost-efficient tools for clinical practice.
